# Q&A: Morphological insights into evolution

**DOI:** 10.1186/s12915-017-0425-z

**Published:** 2017-09-15

**Authors:** Neal Anthwal, Abigail S. Tucker

**Affiliations:** Department of Craniofacial Development and Stem Cell Biology, King’s College London, Floor 27 Guy’s Tower, Guy’s Hospital, London Bridge, London, SE1 9RT UK

## Abstract

In this question and answer article we discuss how evolution shapes morphology (the shape and pattern of our bodies) but also how learning about morphology, and specifically how that morphology arises during development, can shed light on mechanisms that might allow change during evolution. For this we concentrate on recent findings from our lab on how the middle ear has formed in mammals.

## How does evolution help us understand morphology?

**Fig. 1.**
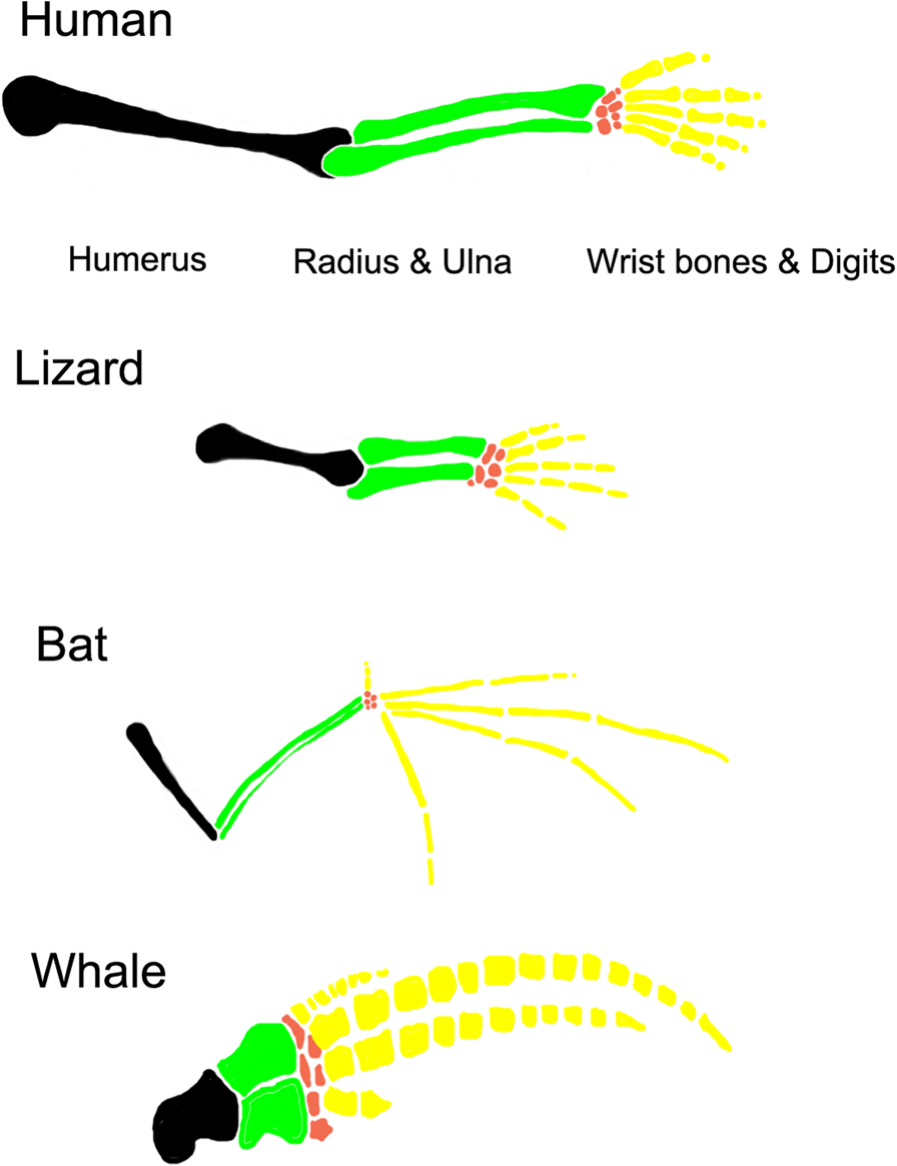
Comparative anatomy of vertebrate limbs. The general skeletal structure of vertebrate limbs is similar in each species, despite large differences in individual bone size and shape reflecting the different functions

Evolution is key to understanding why we look like we do: it can explain why humans have four limbs each with five digits, two forward facing camera eyes, and a mouth full of teeth of different shapes compared to why fruit flies have six limbs plus two wings, two compound eyes, and a proboscis for a mouth. Our anatomy has been slowly shaped over millions of years, and an understanding of evolutionary history can help explain the similar pattern of bones observed in vertebrate limbs. Humans, bats, reptiles and whales evolved from a common ancestor, and the developmental programme to make limbs is shared across these animals and is based on that of this common ancestor. Although the limbs of vertebrates have diverged functionally into the wings of bats, the arms of humans, the forelimbs of reptiles and the fins of whales, they are nevertheless homologous: the general skeletal structure is similar in each, despite large differences in individual bone size and shape (Fig. 1). In contrast, the common ancestor of humans and fruit flies did not have any limbs, so our limbs and the limbs of the fly are independently evolved and not homologous.

## It might be useful to be able to fly—why don’t we evolve wings on our backs like the fly?

It would be useful, but unfortunately it’s nigh on impossible. This is because the form of an organism is made during its embryonic development by following developmental programmes encoded in our genes. These programmes need to be adjusted to change anatomy, and adjustments can only be made on what is already there. Any changes made in earlier developmental programs will have effects on all later programs. Therefore, the possibilities of form (known as phenotype) encoded by the genes (or genotype) are not infinite and form can only change by tinkering with what’s already there. This is called developmental constraint [1]. Insect wings are likely to have evolved from appendages on the exoskeleton of their ancestors that are absent in our lineage, so they cannot be altered to form wings. Although in principle one might evolve to fly in a different way—bats and birds have both independently evolved wings from their forelimbs, what we call convergence—in this case other constraints are in operation. To evolve wings like those of bats, we would have to lose the current function of our hands and arms, which seems an unlikely evolutionary path to take. Other constraints would also be in operation—for example the power required for flight, given the typical human’s weight, would be more than could be generated by our pectoral muscles. When the bones of vertebrates that fly are studied it is clear that they have undergone adaptations to allow flight, with the evolution of hollow or very slender bones.

## What can we learn about evolution by studying morphology?

Morphology is a very useful way of understanding evolutionary processes. Charles Darwin famously noticed differences in beak morphology of Galapagos finches, which helped inform his theory of natural selection and the ‘Origin of species’. Recently the developmental programs underlying shape variation in Darwin’s finches have begun to be understood, with key gene networks—involving *Bmp4*, *calmodulin*, *β-catenin*, *Tgfbr2* and *Dkk*—having been demonstrated to control the size and shape of the beak. Strikingly finch-like beaks could be induced in chick embryos by manipulating these signaling pathways [2]. Understanding morphology, and how that morphology is created in the embryo (developmental biology), can illustrate how it is possible to modify structures and thereby suggest mechanisms that may underlie evolutionary change (evodevo).

## Does this mean that understanding morphology can only tell us about small changes that make species different to each other within groups of animals?

No, while the above examples are compelling examples for the importance of morphological change at the micro level, morphology can be very useful in understanding changes that gave rise to different groups of animals, i.e. evolution at the macro level. For example in our lab we are interested in the morphological and developmental changes giving rise to the evolution of mammals. This work involves comparing embryonic development with the fossil record.

## How can we study mammalian evolution through morphology?

To understand mammalian evolution we need to be able to accurately identify what defines a mammal—but this is somewhat difficult, especially in evolutionary history as observed in the fossil record. Most of the specialisations mammals have are shared by other groups, and so are not on their own sufficient to identify a mammal. Mammals belong to the aminote clade—tetrapod vertebrates that protect their developing embryos—either in an egg or in the mother—in a membrane called the amnion. Other amniotes include the birds and reptiles, and one needs to be able to distinguish mammals from their amniote relatives. While almost all mammals are warm-blooded (the naked mole rat is a possible exception) so are birds, so this can’t be used as a defining feature. It is likely that the common ancestor of mammals and birds was cold blooded, so the presence of endothermy in these two groups is another example of convergent evolution. Most mammals have live births; however, some reptile species such as *Zootoca vivipara* and *Pseudemoia entrecasteauxii* also give birth to live young, while the extant monotreme species (the platypus and two echidna species) lay eggs but are still mammals. All mammals produce milk and most have fur, but these features are not useful since they are not usually preserved in fossils. However, a useful defining feature to identify mammals and distinguish them from other amniotes like reptiles and birds is a specialised middle ear and jaw joint—and this is often easier to find in the fossil record.

## You mentioned the middle ear—what’s the difference between the middle ear in reptiles, birds and mammals?

The ears of reptiles, birds and mammals are made up of three components. These are the outer ear through which sound in the form of vibrating air enters the head, the inner ear in which sound is converted into neuronal signals by vibration of hair cells lining the cochlea, and the middle ear that sits between the two structures.

**Fig. 2.**
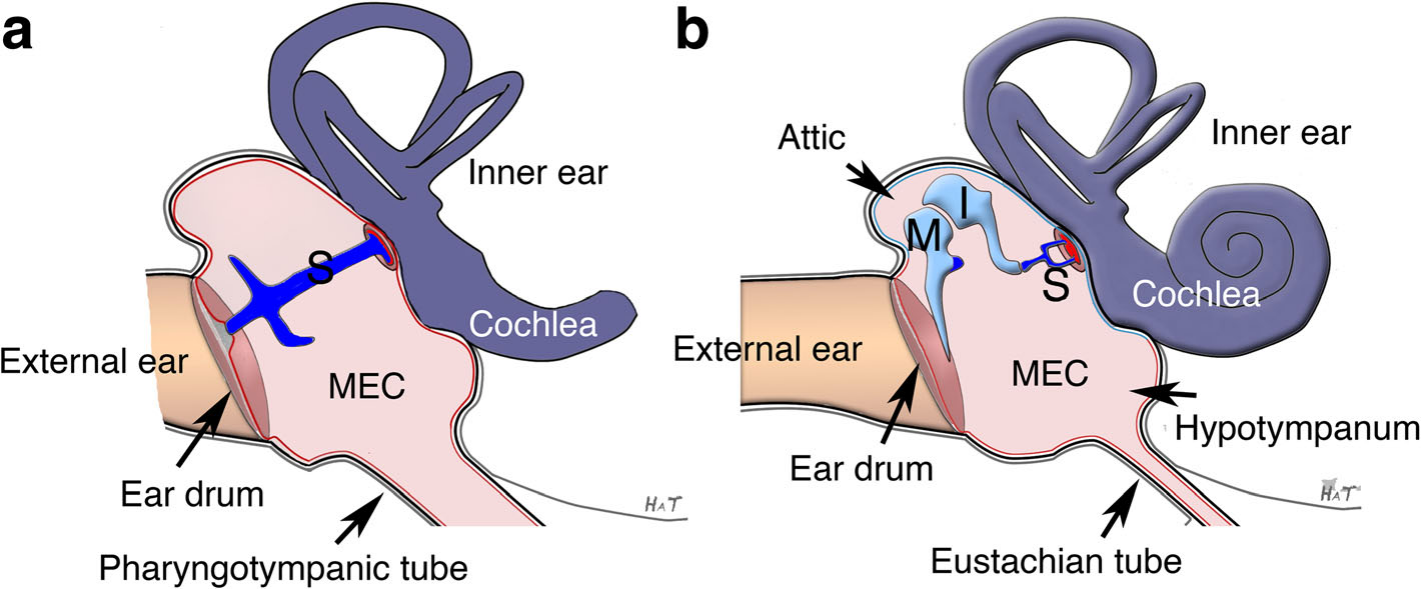
Schematics of a sauropsid (bird, lizard) and mammal ear. In sauropsids (**a**) sound is transmitted from the ear drum to the sensory cells of the cochlea via a single bone, the stapes (S) in the middle ear cavity (MEC). Mammals (**b**) have two extra bones, the malleus (M) and incus (I). Reproduced from [3]

The middle ear is an impedance matching apparatus that facilitates the transmission of sound from the air (low impedance) to the liquid filled inner ear (high impedance). The middle ear consists of the tympanic membrane (ear-drum) for sound capture that is connected to a membrane window into the inner ear via small bones called ossicles. In birds and reptiles there is a single ossicle, called the stapes or columella, whereas mammals have a chain of ossicles, the malleus, incus and stapes (Fig. 2) [3]. In both cases the middle ear ossicle or ossicles are in an air-filled cavity that allows for free vibration and transfer of sound to the inner ear. In whales and aquatic mammals, this air-filled cavity is still present but in addition to sound transfer through the three ossicles, bone and soft tissue conduction occurs through the lower jaw to aid with underwater hearing. A more extreme reliance on bone conduction is observed in snakes. Here the middle ear cavity has been lost and is filled with tissue that surrounds the stapes. The tympanic membrane and external ear are absent and instead sound is detected as vibrations by the lower jaw [3].

## Why does the middle ear differ between mammals and other amniotes?

The extra ossicles of the mammalian middle ear have a surprising origin. The common amniote’s ancestor did not have a tympanic ear—that is to say they had no tympanic membrane or air filled middle ear—and sound was heard by the vibration of bones embedded in tissue connected to the inner ear. In the mammalian lineage of mammal-like reptiles, changes in the jaw musculature and teeth resulted in the evolution of a new jaw articulation (the temporomandibular joint; TMJ) between the squamosal and dentary bones. This new jaw joint appears to have aided stabilisation of the jaw and initially worked together with the original primary jaw joint, located between the quadrate in the cranial base and articular in the mandible. The fossil record reveals examples of mammal-like reptiles, such as Morganucodon, which used both joints to articulate its jaw. The increased efficiency of the new jaw joint allowed the primary joint to become less integrated into the jaw over time, and as a consequence the bones of the jaw reduced in size and were freed up for a new role in hearing. Eventually the primary jaw joint separated completely from the lower jaw. This final separation gave rise to the definitive mammalian middle ear, with the articular being homologous to the malleus, and the quadrate to the incus. The two extra ossicles in the mammalian ear were therefore repurposed from the jaw joint of reptiles—a rather remarkable change in function.

## What is the evidence for this?

The evidence for the transition of the primary jaw joint into the middle ear takes three main forms. Firstly, the fossil record of the transition is remarkably complete and we are able to follow the formation of the TMJ and middle ear ossicles though a wide range of mammalian ancestors known as cynodonts. Secondly, embryology and developmental biology have revealed the mandibular origins of the new parts of the middle ear in mammals. In fact, it was the embryology carried out by Reichert and Gaupp in the 19^th^ and early 20^th^ centuries that first demonstrated the homology between the mammalian middle ear and non-mammalian jaw articulation. Thirdly, we can study marsupials. Marsupials, such as opossums and kangaroos, are born very early in development, before the bones of the jaw are fully formed, yet the young pups need to suckle. They therefore use their middle ear bones, which are still attached to the jaw at this stage, to feed. Once the mammalian jaw joint has formed, the ossicles then revert to a role in hearing. The change from a role in feeding to hearing, mimicking the transformation observed during evolution, can therefore be followed in a living animal.

## You said that the embryology was done over a century ago—what’s the modern take on this problem?

We have recently been using modern developmental biology techniques to try and understand the mechanism of this evolutionary change. Specifically we looked at the cellular and molecular mechanism of the final separation of the ear from the jaw, a developmental process that mirrors evolution. In doing so we were able do demonstrate that a group of cells called clasts are recruited to break down a structure called Meckel’s cartilage that joins the malleus in the ear to the mandible in the lower jaw. In mice the ear and jaw are still physically attached to each other at birth but a wave of clast cell recruitment to this region a few days after birth leads to their separation. In mice with a mutation in *cFos* these clast cells fail to form, and as a result Meckel’s cartilage does not break down, but instead ossifies, and thus forms a hard connection between the jaw and ear. This is similar to the morphology of cynodonts, and so this mutant copies the long extinct cynodont anatomy in a modern mammal [4]. The recruitment of clast cells to this part of Meckel’s cartilage may therefore have been an important step in the isolation of the ear from the jaw, to create the definitive mammalian ear. We were able to confirm that the *Tgf-beta* signalling pathway played an important role in the separation of the ear from the jaw [5]. Furthermore, our evidence also suggests that placental mammals and marsupial mammals have slightly different Meckel’s cartilage breakdown mechanisms, and so may have independently acquired the final step of middle ear evolution.

## Why should I care about evolution and morphology?

An evolutionary insight into morphology can offer ways of understanding some human disorders and diseases. For example one of the most common human developmental disorders is a limb with fewer than five digits. When the limb anatomy of these affected individuals was compared with birds and amphibians that naturally have fewer than five digits, a high degree of similarity was found in the arrangement of muscular attachment to the skeleton [6]. The development of organisms from these phylogenetic classes could therefore offer insights into the basis of the human conditions, and the genetics of the human conditions could inform the understanding of digit evolution.

A further example lies in the middle ear, and the spread of middle ear infection (otitis media). In mammals the epithelium in the lower regions of the cavity is derived from a part of the early embryo called the endoderm, while the remainder, like the large part of the ossicles themselves, is formed by another group of early embryonic cells called the neural crest [7]. This dual origin appears to be unique to mammals and allowed for the creation of an air-filled cavity around the three-ossicles in the middle ear. The endoderm-derived epithelium is complex and covered in cilia while the neural crest-derived epithelium is simpler and unciliated. The two epithelia respond differently to damage, and regions adjacent to the neural crest-lined part of the middle ear (the cochlea and mastoid) are more susceptible to complications due to the spread of middle ear infections, compared to parts of the cavity lined by endoderm. The pattern of spread of ear injections therefore only makes sense in the context of how the ear develops and why it formed in that way during evolution. Understanding how a structure evolved, and how structures are linked during evolution and development, can therefore shed light on why and how abnormalities arise.

## References

[1] Smith JM, Burian R, Kauffman S, Alberch P, Campbell J, Goodwin B (1985). Developmental constraints and evolution: a perspective from the Mountain Lake Conference on Development and Evolution. Q Rev Biol.

[2] Mallarino R, Abzhanov A (2012). Paths less traveled: evo-devo approaches to investigating animal morphological evolution. Annu Rev Cell Dev Biol.

[3] Tucker AS (2017). Major evolutionary transitions and innovations: the tympanic middle ear. Philos Trans R Soc Lond B Biol Sci.

[4] Anthwal N, Urban DJ, Luo Z-X, Sears KE, Tucker AS (2017). Meckel’s cartilage breakdown offers clues to mammalian middle ear evolution. Nature.

[5] Urban DJ, Anthwal N, Luo Z-X, Maier JA, Sadier A, Tucker AS (2017). A new developmental mechanisms for the separation of the mammalian middle ear ossicles from the jaw. Proc R Soc B Biol Sci.

[6] Diogo R, Smith CM, Ziermann JM (2015). Evolutionary developmental pathology and anthropology: A new field linking development, comparative anatomy, human evolution, morphological variations and defects, and medicine. Dev Dyn.

[7] Thompson H, Tucker AS (2013). Dual origin of the epithelium of the mammalian middle ear. Science.

